# Nanoparticle Biomolecular Corona-Based Enrichment of Plasma Glycoproteins for N-Glycan Profiling and Application in Biomarker Discovery

**DOI:** 10.1021/acsnano.1c09564

**Published:** 2022-03-28

**Authors:** Duong N. Trinh, Richard A. Gardner, Alessandro N. Franciosi, Cormac McCarthy, Michael P. Keane, Mahmoud G. Soliman, James S. O’Donnell, Paula Meleady, Daniel I. R. Spencer, Marco P. Monopoli

**Affiliations:** † Department of Chemistry, Royal College of Surgeons in Ireland, University of Medicine and Health Sciences, Dublin 2, Ireland; ‡ Ludger Ltd., Culham Science Centre, Abingdon, Oxfordshire OX14 3EB, United Kingdom; § UBC Faculty of Medicine, Department of Respiratory Medicine, University of British Columbia, Vancouver, British Columbia V6Z 1Y6, Canada; ∥ Department of Respiratory Medicine, St. Vincent’s University Hospital, Dublin 4, Ireland; ⊥ School of Medicine, University College Dublin, Dublin 4, Ireland; # Physics Department, Faculty of Science, Al-Azhar University, Nasr City 11884, Cairo, Egypt; ¶ Irish Centre for Vascular Biology, School of Pharmacy and Biomolecular Sciences, Royal College of Surgeons in Ireland, University of Medicine and Health Sciences, Dublin 2, Ireland; + School of Biotechnology, Dublin City University, Dublin 9, Ireland

**Keywords:** N-glycosylation, glycan profiling, protein corona, biomarker, lung cancer

## Abstract

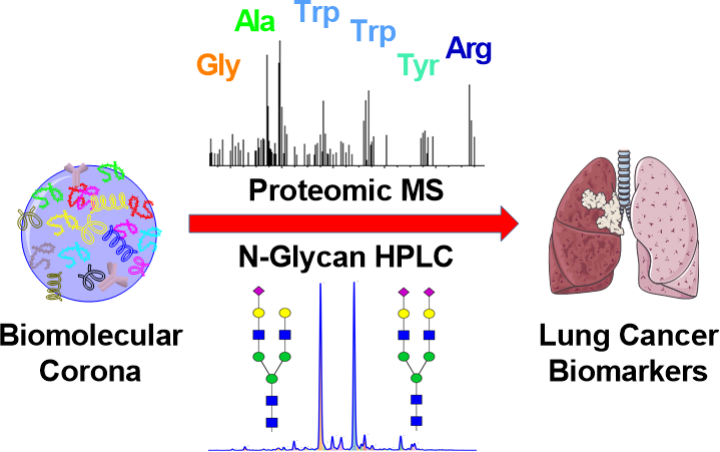

Biomolecular corona formation has emerged as a recurring and important phenomenon in nanomedicine that has been investigated for potential applications in disease diagnosis. In this study, we have combined the “personalized protein corona” with the N-glycosylation profiling that has recently gained considerable interest in human plasma biomarker discovery as a powerful early warning diagnostic and patient stratification tool. We envisioned that the protein corona formation could be exploited as an enrichment step that is critically important in both proteomic and proteoglycomic workflows. By using silica nanoparticles, plasma fibrinogen was enriched to a level in which its proteomic and glycomic “fingerprints” could be traced with confidence. Despite being a more simplified glycan profile compared to full plasma, the corona glycan profile revealed a fibrinogen-derived glycan peak that was found to potentially distinguish lung cancer patients from controls in a pilot study.

## Introduction

When in contact with biological fluids, the surface of nanoparticles (NPs) is spontaneously covered by a selected group of biomolecules including metabolites, lipids, and especially proteins to form a “biomolecular corona”.^1−3^ The corona usually consists of “hard” and “soft” components that have a high affinity toward the NPs’ surface. While the former is the inner tightly bound layer, the latter contains loosely bound molecules on top of the hard corona.^4^ Until now, studies on the biomolecular corona were mostly relevant to nanomedicine and nanotoxicity where it has been shown that in biological milieus, the pristine NP’s surface was fully covered by the corona and it governed the subsequent bionano interactions with cellular receptors.^1,5,6^ Recent studies have reported that the corona protein composition can be affected by the biomolecular composition of the exposing media.^7^ Furthermore, the biomolecular corona obtained from different biological fluids could distinguish healthy individuals from people with disease states, highlighting the specificity and complexity of the paradigm but also showing opportunities in discovery biomarker applications.^8−11^ Until now, most studies have focused on the detection of the protein composition, and the glycan component of the corona remains mostly unexploited, mainly due to the complexity of the glycan characterization methods and the lack of protocols.

**Figure 1:**
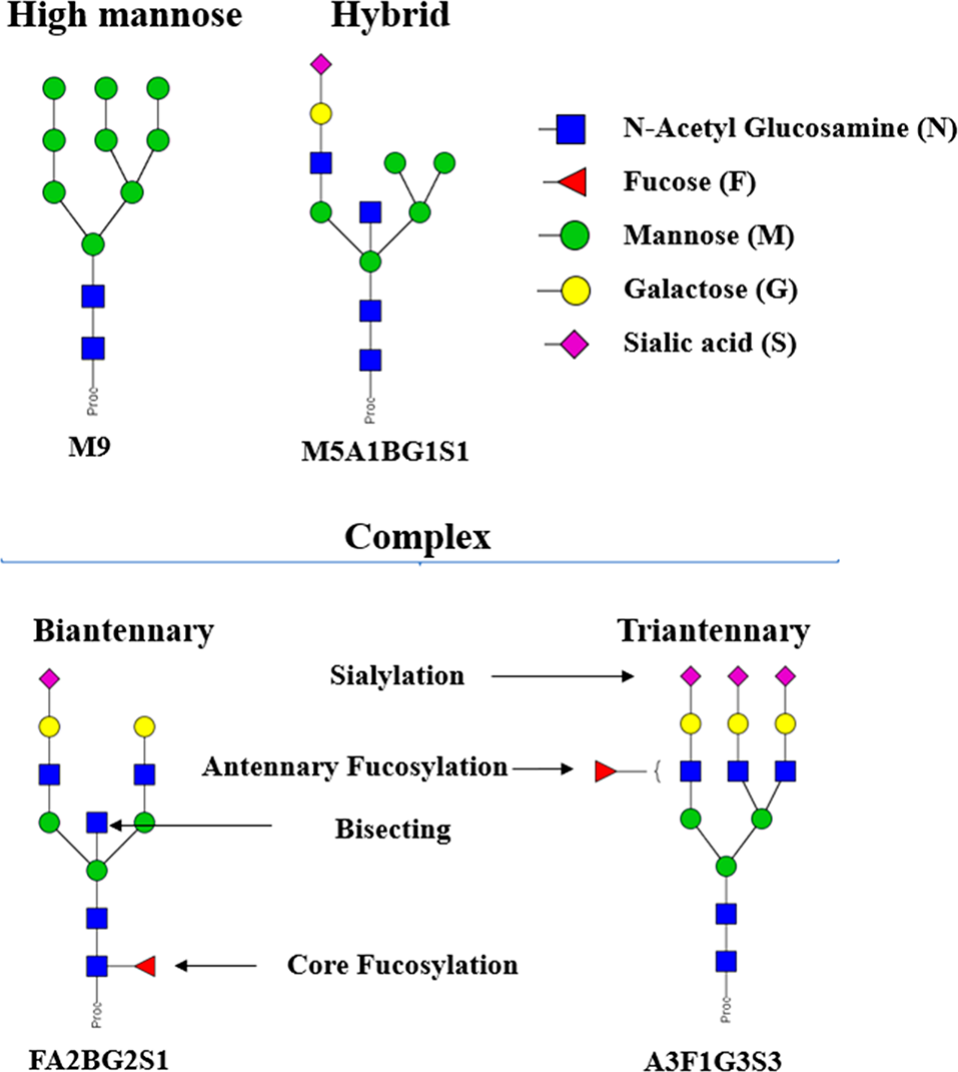
N-glycan types and the system of symbols used in this study for glycans. N-glycans can be grouped into high mannose, hybrid, and complex types. Complex glycans can have up to 4 branches. The glycans were named following the Oxford Notation: antenna (A), bisecting *N*-acetylglucosamine (B), fucose (F), mannose (M), galactose (G), and *N*-acetylneuraminic acid (S).^19^ The glycan structures were depicted following the Consortium for Functional Glycomics (CFG) notation.^20^

N-glycosylation is one of the most important and intricate post-translational modifications of proteins, with respect to the complexity of the added carbohydrates and the magnitude of the cellular machinery devoted to synthesis and modulation.^12^ Human plasma N-glycans can be classified into three groups that share the same core structure: high-mannose, complex, and hybrid glycans (Figure 1).^13^ With the development of standardized high-throughput characterization techniques, unknown and disease specific glycan signatures have been detected and used as early warning biomarkers for risk stratification, diagnosis, and prognosis.^14−16^ In spite of the heterogeneity and diversity, selective changes in the N-glycosylation have been shown to be specific to cancer and inflammation.^17,18^

Blood plasma is a complex but convenient and information-rich sample for biomarker discovery, containing more than 10 000 proteins, whose concentrations vary by more than 10 orders of magnitude. Furthermore, the top 12 most abundant proteins, including albumin, immunoglobulin G (IgG) and transferrin, account for more than 90% of the total plasma protein mass.^21−23^ Meanwhile, a typical upper limit of dynamic range in commercial mass spectrometry (MS) is much lower, about 5 orders of magnitude, which leads to inevitable information loss for low-abundance proteins.^24^ The presence of glycosylation adds another layer of complexity to the plasma analysis as N-glycosylation profiling introduces further analytical challenges.^25^ As a result, different enrichment strategies have been developed to reduce plasma complexity, including fractionation, immunoaffinity chromatography, and glycoprotein enrichment (lectin affinity, hydrazide, and HILIC chromatography).^26,27^ It is worth mentioning that the relative abundances of plasma proteins can be very different from those of plasma N-glycoforms. While plasma proteins with very low abundances have been the main target of proteomic biomarker discovery, glycan profiles of fairly abundant glycoproteins could also provide useful information about the disease state. In fact, many glycan-based biomarker studies have focused on the glycosylation of IgG and acute phase proteins.^28,29^ These proteins are mainly separated from plasma using immunoaffinity chromatography methods, which are protein-specific but can be costly for a biomarker screening, routine, or large-scale cohort analysis.

Our previous study showed that the biomolecular corona composition of silicon dioxide (silica) NPs is highly correlated to the percentages of plasma proteins during the incubation step.^30^ In particular, the presence of fibrinogen and apolipoprotein A1 (apoA1) increased in the corona in a protein-deprived environment while they became progressively displaced by other proteins (*e.g*., histidine-rich glycoprotein, kallikrein B, and plasminogen) when the NPs were exposed to higher plasma concentrations. Although the biomolecular corona has emerged as a promising tool for disease stratification, no study has yet exploited the glycan profiles of corona proteins for this purpose. In this study, we demonstrate a platform to selectively enrich fibrinogen from human plasma for glycan profiling, with an acute phase glycoprotein playing an important role in many physiological processes, and develop a platform to characterize the protein and glycan components.^31^ A pilot study on a lung cancer cohort was performed to investigate the potential of using the proteomic and, especially, glycan profiles of the corona for disease diagnosis.

## Results and Discussion

### Plasma Protein Enrichment with Silica Nanoparticles

**Figure 2:**
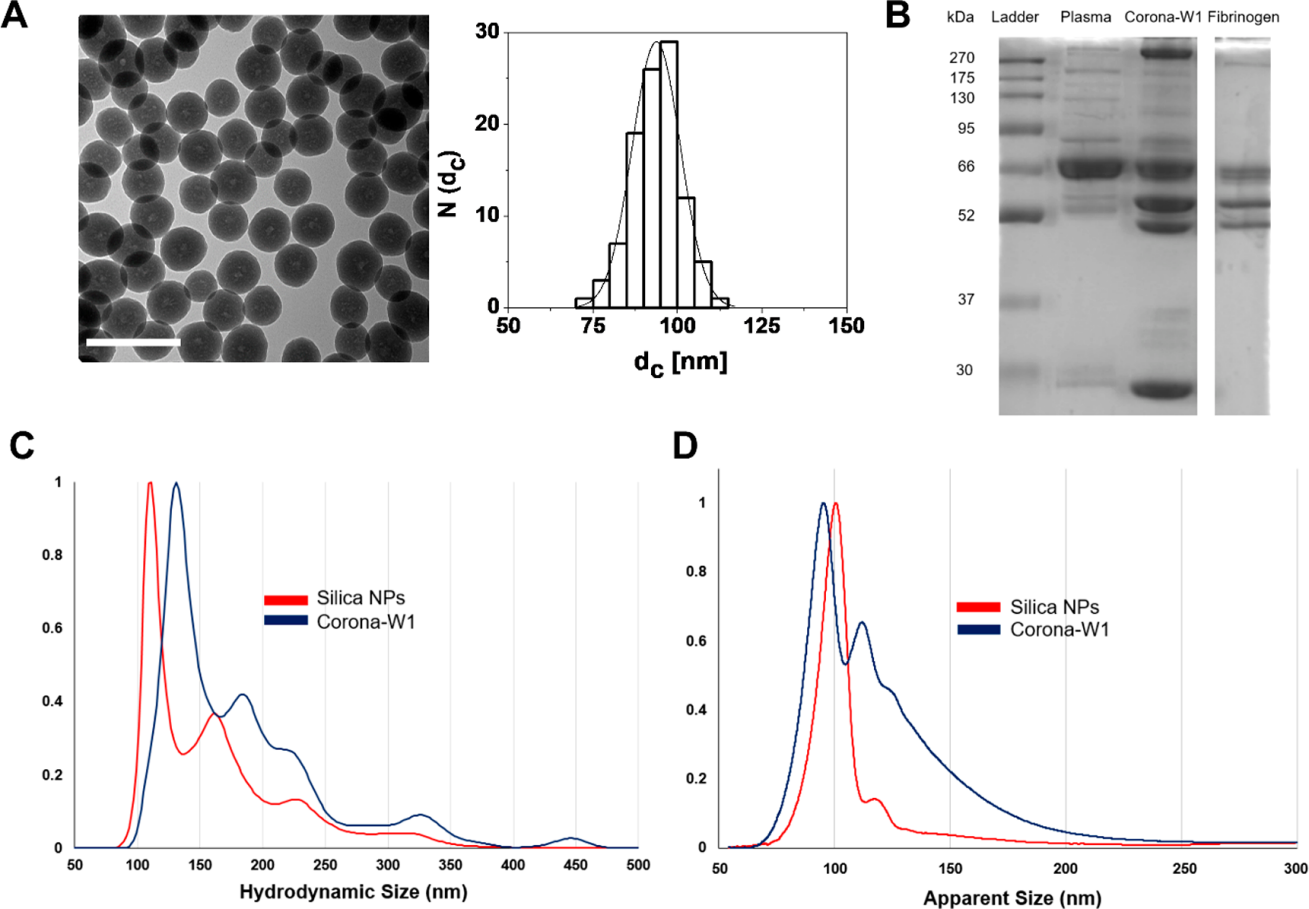
Characterization of the silica NP and its biomolecular corona (1 wash, W1) at the plasma/NP ratio of 1.98. (A) TEM images of silica NPs and their corresponding size distribution histograms, plotted as number of NPs *N*(*d*_c_) that have a core diameter of *d*_c_ = 93.7 ± 7.1 nm. The scale bars correspond to 200 nm. (B) The protein pattern of the corona in comparison with full plasma and fibrinogen. (C) NTA hydrodynamic size distributions of pristine silica NPs and silica corona. The data are shown as the average of three measurements. The peaks were normalized against the tallest peaks (highest particle concentration) that had a value of 1. (D) DCS size distributions of pristine silica NPs and silica corona. The data were shown as relative weight particle size distribution. The tallest peak (highest weight value) had a value of 1, and all other particle size peaks were then normalized against this base peak to give a relative weight distribution.

The size distributions of the silica pristine NPs and the coronas were characterized by different methods, including transmission electron microscopy (TEM), dynamic light scattering (DLS), nanoparticle tracking analysis (NTA), and differential centrifugal sedimentation (DCS). The particles were monodispersed with the TEM core size of 93.7 nm (Figure 2A), a hydrodynamic size of 114.2 nm, and a polydispersity index (PDI) of 0.03 (Table 1). The presence of hydroxyl groups on the NP surface rendered the surface charge negative at the physiological pH (zeta potential of −45.4 mV in phosphate buffer 1 mM, pH 7.4).

**Table 1:** Hydrodynamic and DCS Sizes of Pristine Silica NPs and Silica Coronas after 1, 2, and 3 Centrifugal Washes (W1, W2, and W3)^a^

	DLS ± SD(*n* = 3)		NTA		DCS ± SD(*n* = 4)	
sample	*Z*-average	PdI	main peak	2nd peak	main peak	2nd peak
					corona thickness	
Silica NPs	114.2 ± 1.2	0.03 ± 0.02	111.0	161.0	102.1 ± 0.7	118.9 ± 1.4
					0	
Corona-W1	175.1 ± 0.9	0.14 ± 0.02	131.0	183.0	93.2 ± 0.5	111.4 ± 1.0
					15.9 (14.6–17.3)	
Corona-W2	174.3 ± 1.6	0.16 ± 0.01	127.0	171.0	93.4 ± 0.4	111.3 ± 1.2
					15.4 (14.4–16.4)	
Corona-W3	169.8 ± 2.0	0.15 ± 0.02	125.0	179.0	93.5 ± 1.0	111.0 ± 1.9
					15.1 (12.7–17.8)	

a DLS measurements (*n* = 3). SD: standard deviation. NTA measurements (*n* = 3), the peaks were reported after merging three technical replicates. DCS measurements (*n* = 4). The sizes of the main and second peaks are shown for NTA and DCS. All sizes were in nanometers.

Silica corona complexes were prepared and isolated by previously developed methods.^32^ The size distributions of the silica biomolecular coronas after different numbers of washes were measured to obtain the information about the NPs’ dynamic corona (1 wash, W1) and strongly bound one (2 and 3 washes, W2 and W3) and whether the washing process affected the particle aggregation state (Table 1).^33^ NTA analysis shows a shift of the NP-corona W1 particles compared to the pristine ones, as a result of the protein deposition. (Table 1, Figure 2C). The hydrodynamic size distributions of the samples with the increasing number of washes were similar to each other, with only a slight decrease in size of the main peak, indicating that the washing steps had little effect on the NP colloidal stability (Figure S1A). Meanwhile, in the DCS data, the major peak of the corona-W1 (93.2 nm) was shifted to an apparent lower size (shift to the left) in relation to the pristine silica (102.1 nm, Figure 2D) as a result of the removal of the loosely bound corona proteins.^34^ The calculated DCS shell thickness of the main peak also decreased when comparing all corona samples, from 15.9 nm (corona-W1) to 15.1 nm (corona-W3) (Table 1), indicating that the washes lead to a small progressive protein corona loss with the increase of the washes.

SDS-PAGE gel shows that the corona was highly enriched in fibrinogen as a triple band between 45 and 70 kDa, which were shared with the purified fibrinogen (Figure 2B). However, a protein band of 30 kDa was also detected, indicating the presence of other proteins. The collected supernatants after each washing step were also analyzed, and it can be seen that the first wash resulted in the washing of loosely bound proteins of a wide range of sizes. (Figure S1C). After that, the second and third washes gradually removed the main corona proteins as their intensity was gradually decreased. Overall, the results indicate that there is only a subtle and qualitative difference between the coronas obtained after different centrifugal washes and the overall corona composition remained unchanged. Hence, we decided to focus on the silica corona with only one wash in order to ensure a high sensitivity of the signal.

To further evaluate the fibrinogen corona enrichment, and to measure the potential impact of a different source of blood plasma, we assessed the reproducibility using eight plasma samples obtained from healthy donors to form the corona, where we kept the same ratio between the NP surface area and total protein in the media. Overall, fibrinogen could make up about 40% of the protein signal coverage and with a similar protein pattern across the samples (Figure S2A). Meanwhile, in the protein profile of full plasma, the single band at 66 kDa of albumin was dominant, accounting for nearly 50% of the total protein signal (Figure S2B). The results indicate that the protocol was consistent in the enrichment of fibrinogen when using plasma samples obtained from different individuals.

### Proteomic Features of Fibrinogen-Enriched Corona

Shotgun proteomics was performed to further investigate the protein corona composition of the silica corona. A total of 291 proteins were identified in the corona sample, and in addition to fibrinogen, apoA1 and apoB100 were highly abundant in the corona, which indicates the presence of HDL and LDL, respectively (Table 2). These two apolipoproteins can be observed on the SDS-PAGE gel at 28 kDa band (apoA1) and high MW bands near 270 kDa (apoB100). Interestingly, serum albumin, the most abundant plasma protein of the blood is not in the top 10 abundance of the corona protein composition suggesting a low affinity toward the silica NP surface.

**Table 2:** Top 20 Abundant Proteins in the Corona Ranked by the Summed Peptide Ion Intensity Identified by Mass Spectrometry^a^

silica corona					full plasma^32^	
no.	intensity (log_2_)	Uniprot identifier	protein names	MW (kDa)	protein names	concn (μg/mL)
1	37.3	P02647	Apolipoprotein A-I	30.78	Albumin	4.0 × 10^4^
2	36.8	P02679	**Fibrinogen gamma chain**	49.50	Immunoglobulin heavy constant gamma 1	1.1 × 10^4^
3	36.8	P02675	Fibrinogen beta chain	55.93	Transferrin	2.3 × 10^3^
4	36.2	P02671	Fibrinogen alpha chain	94.97	Immunoglobulin heavy constant alpha 1	2.0 × 10^3^
5	33.8	P04114	**Apolipoprotein B-100**	515.6	Apolipoprotein A-I	1.4 × 10^3^
6	33.2	P04196	**Histidine-rich glycoprotein**	59.58	Alpha-2-macroglobulin	1.4 × 10^3^
7	32.6	P01042	**Kininogen-1**	71.96	Alpha-1-antitrypsin	1.1 × 10^3^
8	32.5	P02649	**Apolipoprotein E**	36.15	Complement C3	9.5 × 10^2^
9	32.1	P06727	Apolipoprotein A-IV	45.37	Haptoglobin	8.8 × 10^2^
10	31.7	P02751	Fibronectin	246.69	Immunoglobulin heavy constant mu	8.0 × 10^2^
11	31.5	P02768	Albumin	69.37	Hemopexin	7.5 × 10^2^
12	31.4	P01024	**Complement C3**	187.15	Apolipoprotein B-100	7.2 × 10^2^
13	31.1	P00748	**Coagulation factor XII**	67.79	Fibrinogen gamma chain	6.7 × 10^2^
14	30.8	D6R934	Complement C1q subcomponent subunit B	26.46	Alpha-2-HS-glycoprotein	6.3 × 10^2^
15	30.6	A0A4W9A917	Immunoglobulin heavy constant gamma 3 (Fragment)	41.22	Alpha-1-acid glycoprotein 2	6.1 × 10^2^
16	30.4	K7ERI9	Apolipoprotein C-I (Fragment)	8.65	Complement factor H	5.0 × 10^2^
17	30.2	P02747	Complement C1q subcomponent subunit C	25.77	Interalpha-trypsin inhibitor	5.0 × 10^2^
18	30.2	P27169	**Serum paraoxonase/arylesterase 1**	39.73	Alpha-1-antichymotrypsin	4.5 × 10^2^
19	30.1	P05155	**Plasma protease C1 inhibitor**	49.76	Ceruloplasmin	4.0 × 10^2^
20	29.8	A0A096LPE2	SAA2-SAA4 readthrough	23.35	Vitamin D-binding protein	4.0 × 10^2^

a For comparison, the top abundant proteins from full plasma are also shown.^35^ Corona proteins carrying N-glycans registered in the Uniprot database are bolded (accessed June 16, 2021; https://www.uniprot.org/).

More than half of these top 20 abundant proteins forming the corona are known to be N-glycosylated, particularly fibrinogen, apoB100, histidine-rich glycoprotein and kininogen-1. ApoA1 (28 kDa) is the most abundant protein in the silica corona, but the protein is known not to carry N-glycans. The enrichment analysis shows that the silica corona contained mainly proteins related to humoral immune response and coagulation processes, accounting for 34.5% and 24.6% of the Gene Ontology (GO) terms, respectively (Figure S3). However, taking into account the top abundance list, the protein groups that likely represent the silica corona proteins are lipoproteins (apoA1, apoB100, apoE, apoA4, and apoC1) and coagulation-related proteins (fibrinogen, histidine-rich glycoprotein, kininogen-1, and factor XII).

### Glycan Profile of Fibrinogen-Enriched Biomolecular Corona

**Figure 3:**
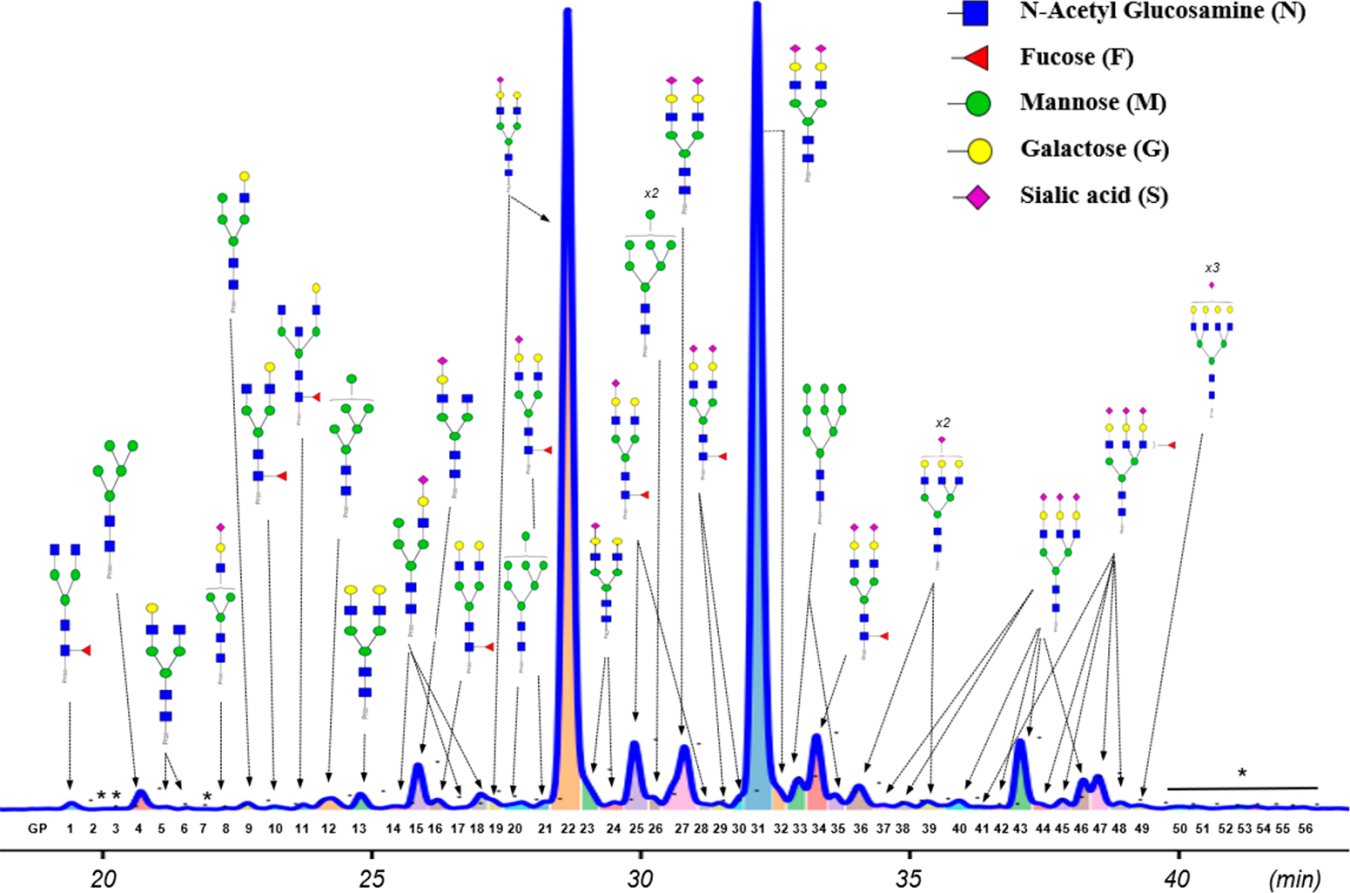
Glycan profile of the silica corona. The glycan linkages of galactose, fucose, and sialic acid are not specified. For a peak with multiple glycan structures detected, only the major structure is shown. *: no glycan characteristic *m*/*z* was registered for these peaks.

To study the glycan profile of the silica corona, we modified a standard protocol using PNGaseF enzyme which cleaves the linkage between the core N-acetylglucosamine (GlcNAc) and the asparagine residue on proteins, releasing all N-glycans except those containing a fucose α1-3 linked to the reducing terminal GlcNAc found in nonhuman species.^36,37^ The released glycans were labeled with a fluorophore before being analyzed by HILIC chromatography coupled with a fluorescence detector and electrospray ionization MS (HILIC-FD-ESI-MS). Using a 70 min HPLC gradient alongside ESI-MS allowed the identification of 56 peaks, 46 of them were assigned with the glycan structures (Figure 3 and Table S2). For comparison, the glycan profile of full plasma containing 59 peaks is shown in Figure S4 and Table S1. It was found that one of the most abundant glycan structures present in the corona’s glycan profile, A2G2S1, can be atributed to the enrichment of fibrinogen. Fibrinogen is a circulating glycoprotein synthesized by the liver hepatocytes, and among its three peptide chains, only β- and γ-chains are N-glycosylated, predominantly with A2G2S1 (52.98%) and A2G2S2 (32.56%).^38^ In the silica corona, A2G2S1 peak accounted for 32.25% of the total N-glycome, a significant increase from only 10.44% in the full plasma profile. On the other hand, the presence of A2G2S2 in the silica corona decreased slightly (from 37.34% to 35.15%) and the abundances of other glycan structures were considerably reduced (Figure S5). Some specific glycan structures that were enriched in the silica corona are shown in Table S3.

**Figure 4:**
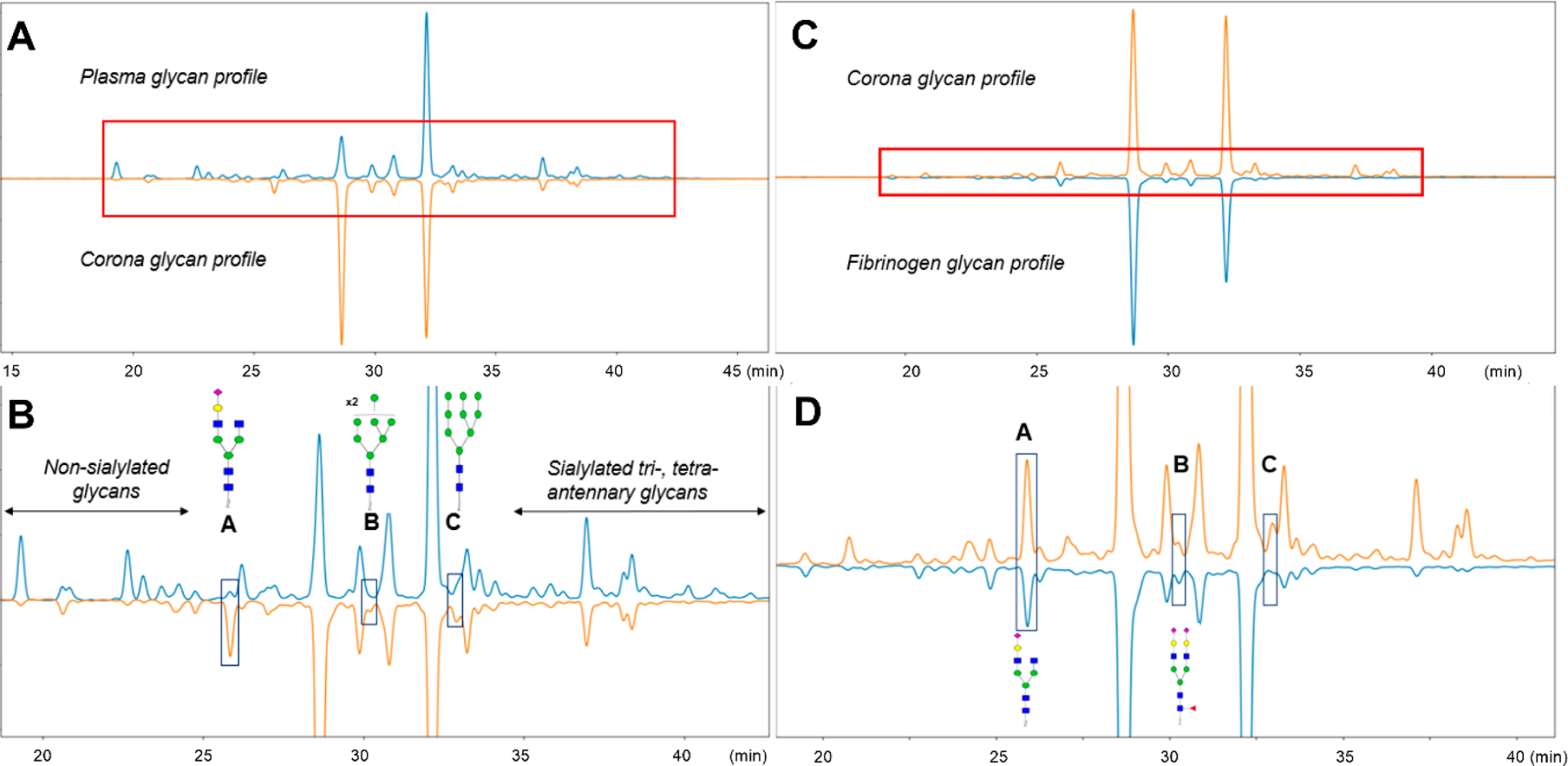
Comparison of the silica corona glycan profile with full plasma (A,B) and fibrinogen (C,D). (A) Chromatograms of released glycans from plasma (up, blue) and the silica corona (down, orange) were normalized to the highest peak intensity (GP_corona_31 in the corona’s chromatogram). (B) Zoomed-in section of the box highlighted in part A. Three areas enclosed by boxes show noticeable differences between the two glycan profiles, containing A2G1S1 (A), coeluted M8 and FA2G2S2 (B), and M9 (C). (C) Chromatograms of released glycans from the silica corona (up, orange) and fibrinogen (down, blue) were normalized to the highest peak intensity (GP_corona_22). (D) Zoomed-in section of the box is highlighted in part C. Boxes A and B contain two shared glycans between the corona sample and fibrinogen control.

It can be seen that the glycan complexity of the corona was lower than that of the full plasma, particularly in the retention time regions of 17–25 min and 35–45 min, which contained nonsialylated biantennary glycans and sialylated tri-, tetraantennary glycans, respectively (Figure 4A,B). The decrease in the peaks detected in these regions could be attributed to the selective plasma enrichment toward a few numbers of highly abundant glycoproteins. Furthermore, GP_corona_15 (region A) containing A2G1S1 was enriched in the corona, while additional glycan peaks were detected in region B (GP_corona_26) showing a peak splitting of M8 and region C (GP_corona_33) showing a peak splitting of M9.

We then compared the glycan profiles of the silica corona with that of fibrinogen obtained from a commercial source. Their glycan profiles were similar, sharing predominant glycans A2G2S1 and A2G2S2, along with others, for example, A2G2 and FA2G2S1 (Figure 4C,D). More importantly, the two corona glycan peaks in regions A (GP_corona_15) and B (GP_corona_26) were also present in the fibrinogen glycan profile, which indicates that enrichment of fibrinogen was the reason behind the emergence of these peaks. Although FA2G2S2 was less abundant than M8 in GP_corona_26, we concluded that this FA2G2S2 isoform of fibrinogen could only be analyzed in the silica corona samples. In contrast, M8 in GP_corona_26 and M9 in box C (GP_corona_33) were not observed in the purified fibrinogen, indicating that these high mannose glycans were linked to other corona proteins.

### Fibrinogen Enrichment Method Applied in Biomarker Discovery of Lung Cancer

Lung cancer is the principal cause of cancer-related death worldwide, causing up to 3 million deaths annually.^39^ It is a complex cancer with different subtypes and stages. Histologically, 80–85% of lung cancers are classified as nonsmall cell lung cancer (NSCLC), while the remaining are small cell lung cancer. The major subtypes of NSCLC are adenocarcinoma, squamous cell carcinoma, and large cell carcinoma.^40^ As the survival rate of lung cancer patients increases significantly if diagnosed early, a noninvasive diagnostic procedure for this disease, particularly a plasma biomarker, is highly sought.^41^

In this pilot study, 25 plasma samples of patients diagnosed with different types of lung cancer, mainly adenocarcinoma (15 samples) and squamous cell carcinoma (7 samples), were processed with silica NPs to enrich fibrinogen and compare with the non-lung cancer group. The sample information, including age, sex, and total plasma protein concentrations determined by a Bicinchoninic acid assay (BCA), is shown in Table 3. For each corona sample, the protein/NP concentration ratio was set to 1.98, which is equivalent to the condition used in the silica corona described above. Both full plasma and corona released glycans were analyzed while quantitative MS was only used for the silica corona samples.

**Table 3:** Cohort Sample Information^a^

feature	non-lung cancer (*n* = 26)	lung cancer (*n* = 25)
age in year (median [IQR])	63 (59–67.5)	72 (66–73)
sex (male/female)	10/16	10/15
total plasma protein concentration in mg/mL (median [IQR])	65.9 (63.4–73.48)	79.62 (75.42–85.11)

a Descriptive information for 26 non-lung cancers and 25 lung cancers. The continuous variables age and total protein concentration are shown as medians and interquartile ranges while the categorical feature sex uses the basic counts.

First, the corona sizes of the cohort samples were characterized by DLS, which is a simple and quick benchtop technique for size measurement. The enrichment method was found to be compatible with the plasma cohort in terms of the colloidal stability as the majority of samples were quite stable with the PdI below 0.25 (Table 4). There was no noticeable difference in the coronas’ sizes between the two groups. It is important to ensure the stability of the samples obtained with the biomolecular corona formation so that variations in the corona’s protein composition and their relative amounts were better controlled.

**Table 4:** DLS Size Summary of the Cohort^a^

feature	non-lung cancer (*n* = 26)	lung cancer (*n* = 25)
*Z*-average size in nm (median [IQR])	194.1 (188.3–202.2)	192.6 (185.3–210.5)
PdI (median [IQR])	0.15 (0.13–0.19)	0.14 (0.11–0.18)

a The colloidal stability of the samples from the two groups are comparable. Hydrodynamic size and PdI are shown as medians and interquartile ranges.

**Figure 5:**
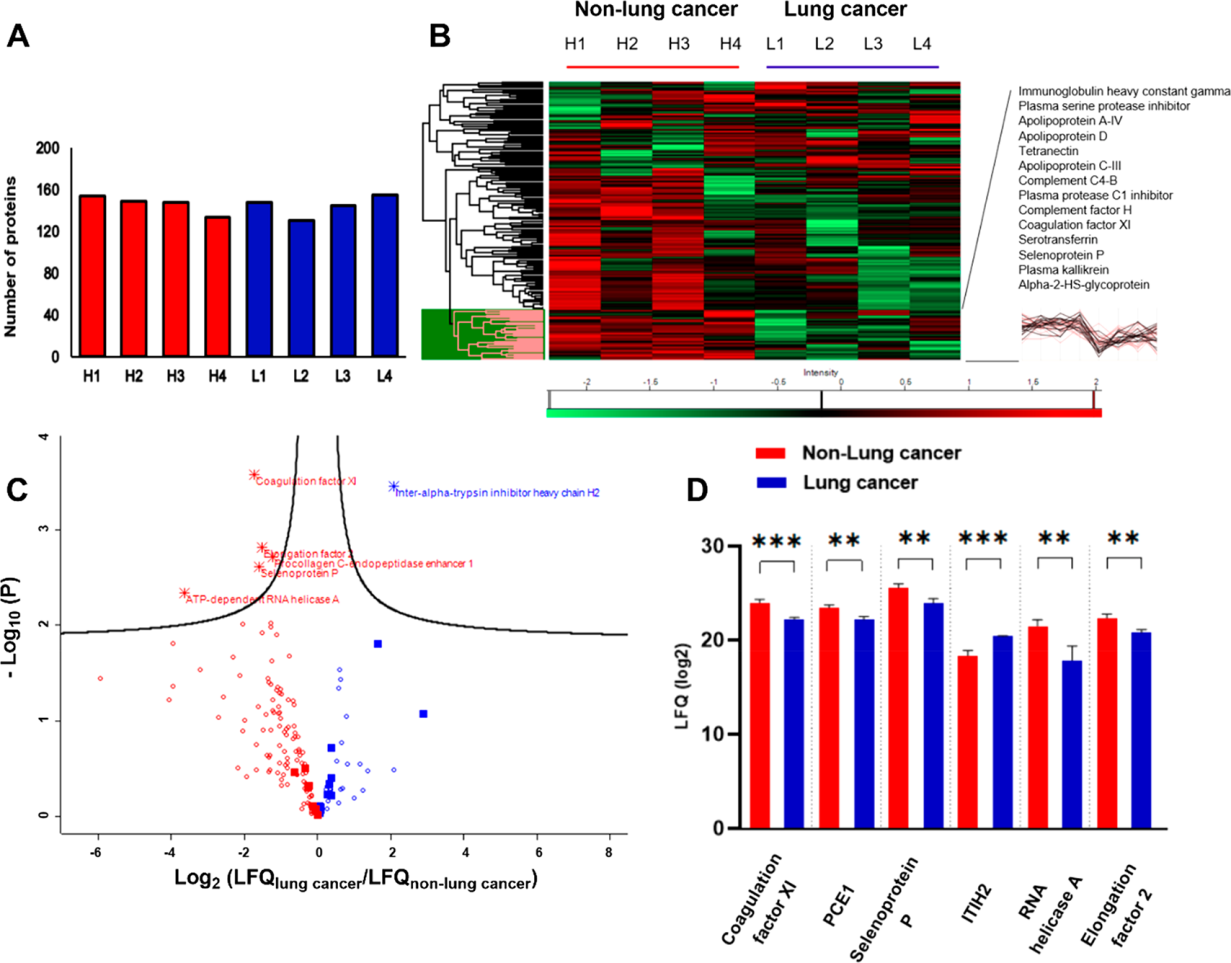
Comparison between the lung cancer (L1, L2, L3, L4) and non-lung cancer samples (H1, H2, H3, H4) by LFQ-MS. (A) Numbers of proteins that met the LFQ normalization criteria (default in Maxquant). (B) Protein heatmap of the samples after *z*-scoring. A protein cluster was expanded with some protein of interest and their intensity profiles (black). (C) Volcano plot with fold-change and *p*-values shows the *t* test result between the two groups. Red: proteins more abundant in the non-lung cancer group. Blue: proteins more abundant in the lung cancer group. Star: 6 proteins with significant difference. Filled square: proteins in the top 20 abundances. (D) LFQ intensity of the proteins with significant differences. Error bars: SD of the mean. *P* values <0.01**; <0.001***.

After that, a label free quantification (LFQ)-based MS strategy was performed in Maxquant to compare the protein abundance between selected samples from each group (*n* = 4). LFQ intensities are normalized median mass spectra intensity values that allow this quantification to be performed with any peptide and protein fractionation while maintaining high accuracy.^42^ There were between 130–155 proteins (out of 291) in each sample that could be used for the intensity-based comparison (Figure 5A). The protein abundance patterns of the two groups shown in the heatmap were found to be quite heterogeneous, but a protein cluster stood out as being enriched in the corona of the non-lung cancer group. This cluster is highlighted in pink with the names and intensity profiles of some protein members (Figure 5B). A multiple *t* test with FDR correction identified six proteins that were significantly different between the two groups: coagulation factor XI, Procollagen C-endopeptidase enhancer 1 (PCE1), selenoprotein P, interalpha-trypsin inhibitor heavy chain H2 (ITIH2), ATP-dependent RNA helicase A, and elongation factor 2. All of them had the fold changes larger than 2 as can be seen in the volcano plot (Figure 5C). The genes encoding interalpha-trypsin inhibitor and selenoprotein P have been reported to downregulate in lung cancer tissue.^43,44^ Interalpha-trypsin inhibitor, whose presence, although at low abundance, could be associated with the abundant lipoprotein HDL in the corona, is N-glycosylated with biantennary glycans.^45^

The glycan peak abundances in the disease and control samples were compared with HappyTools software.^46^ The peak areas were normalized by the total, then were inversed and log-transformed to avoid the constant-sum constraint and reduce the positive skewness.^15^ Various physiological and behavior parameters such as age, sex, body mass index, and several environmental factors, including smoking, have been shown to associate with protein glycosylation.^14^ For both full plasma and corona glycan data sets, the association of peak areas with the age and gender were checked before further analyses and no relationship between them was established (data not shown). As the ages and genders of the individuals between the groups were matched quite well (Table 3), their association with the glycan peaks would be limited. Hence, we decided to not include them into the statistical tests between the two groups. In the full plasma sample analysis, 15 glycan peaks were found to be significantly different between the two groups (Figure S6 and Table S4). As shown in Table 5, glycan peaks carrying sialylated tri- and tetra-antennary glycan structures were increased in the lung cancer samples while different trends were observed for the biantennary glycan structures.

**Table 5:**
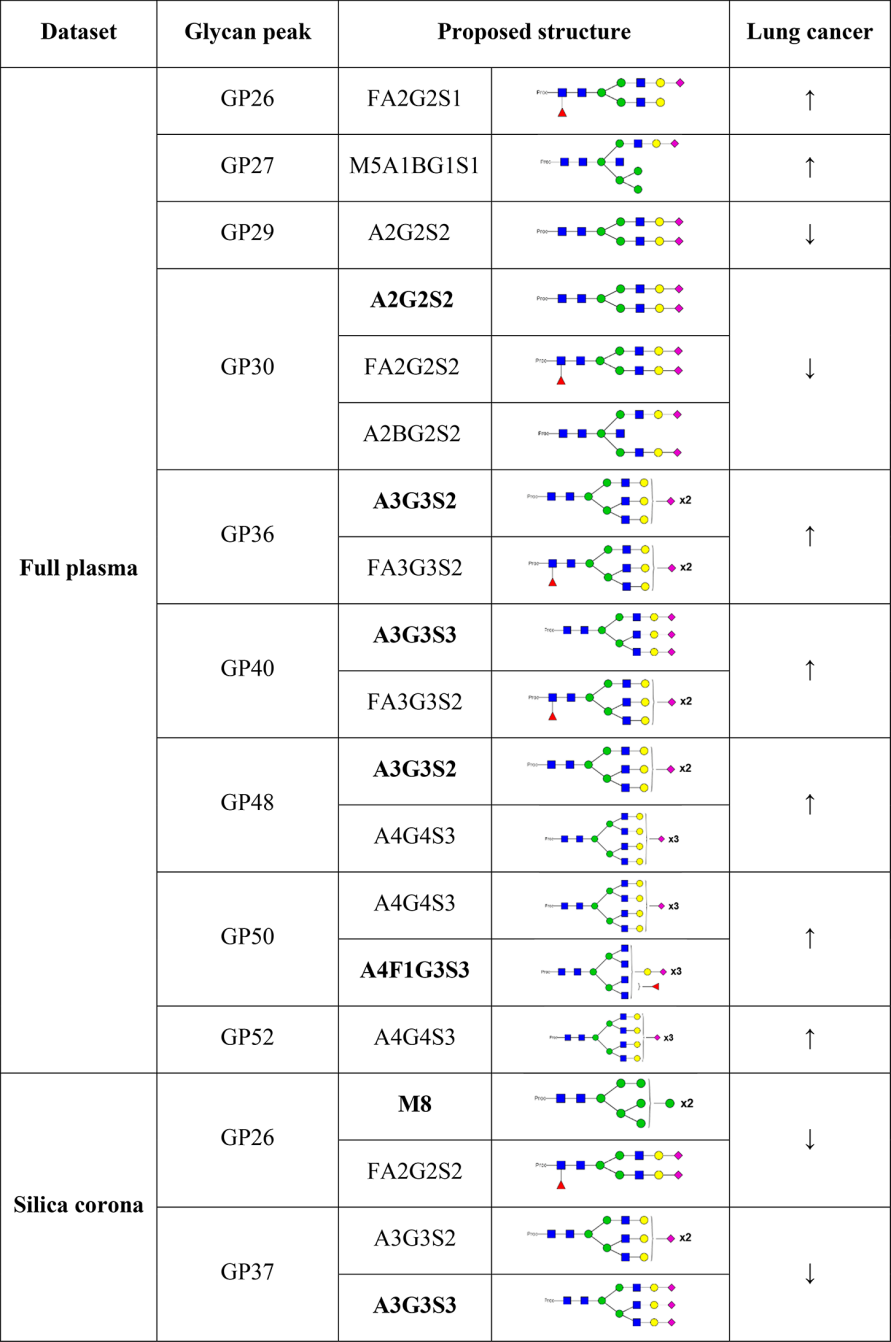
Summary of the Glycan Peaks That Were Found to Significantly Associate with Lung Cancer^a^

a (↓) Decrease, (↑) increase. Major glycans are bolded.

A2G2S2 in the most abundant glycan peak was found to decrease in the lung cancer group, and this glycan is present in various proteins, including transferrin, fetuin, haptoglobin, and also fibrinogen. A statistically differences in peaks containing fucosylated glycans were also detected, and it is in agreement with the literature where increase of inflammation and tumor lead to a dysregulation of the fucosyltransferases.^18^

**Figure 6:**
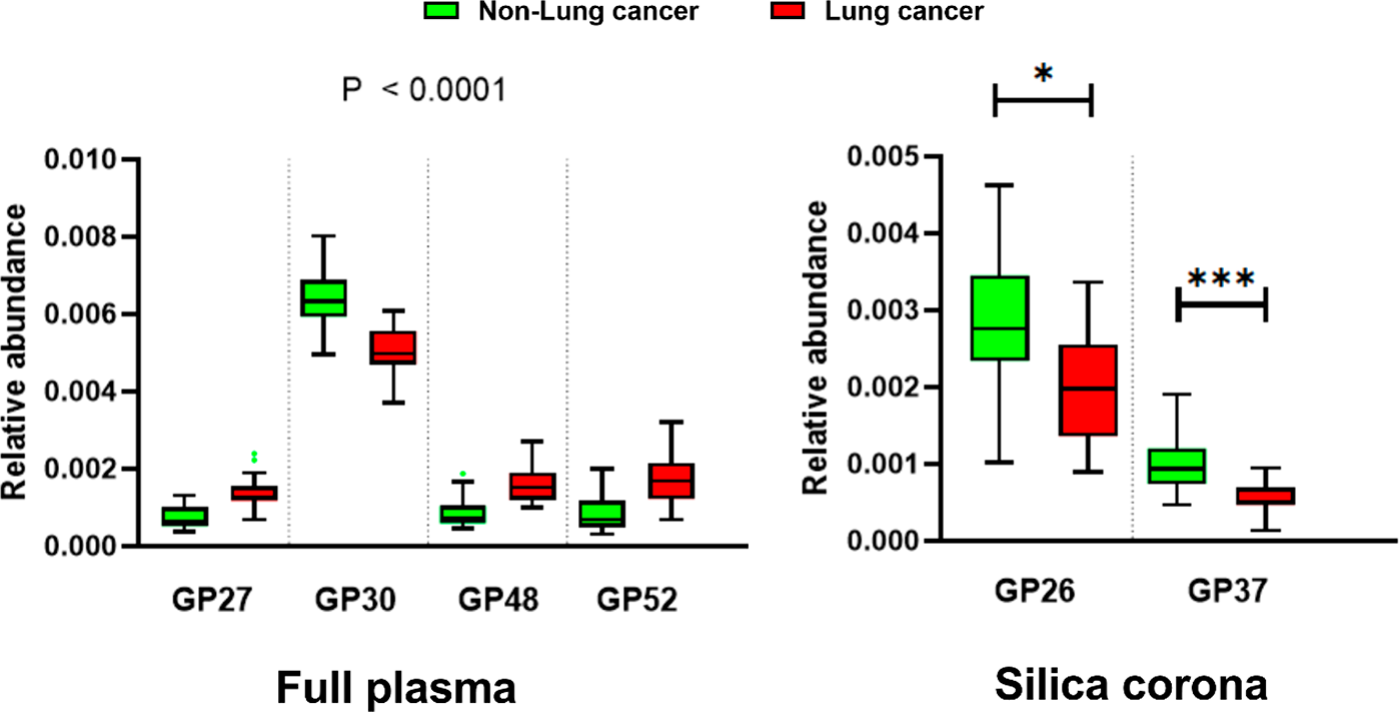
Peaks with significantly different areas between the lung cancer and non-lung cancer groups. Four plasma glycan peaks with the strongest significance are shown. There are only two glycan peaks from the silica corona that were found to be significantly different: GP_corona_26 and GP_corona_37. Relative peak abundance was obtained from HappyTools. Box plots with Tukey whiskers. Adjusted *P* values <0.05*; <0.001***.

On the other hand, in the corona glycan profile analysis, only two peaks were found to be significantly different between the two groups, containing the glycan structures M8 (GP_corona_26) and A3G3S3 (GP_corona_37) (Table S5). The differences of some glycan peaks from both full plasma and corona analyses are shown in Figure 6. It is interesting to observe the decrease in the corona peak containing sialylated triantennary glycan A3G3S3, which was found to increase in multiple glycan peaks in the full plasma analysis (GP_plasma_40, 44, and 48). GP_corona_26 containing M8 and a FA2G2S2 isoform was not available in the full plasma analysis as the peak only emerged in the silica corona profile. As this glycan peak was present in the fibrinogen glycan profile, the finding indicates that the FA2G2S2 glycoform of fibrinogen could be specifically altered in the lung cancer disease state.

In this study, plasma fibrinogen was enriched by using 100 nm silica NPs at a specific plasma/NP ratio for N-glycan profile analysis. The method is simple to incorporate into the glycan profiling workflow, and a high-throughput setup can be established, for example, with silica-coated iron oxide NPs. The enrichment of fibrinogen can be seen in both proteomic and glycomic profiling. Among the top abundant plasma proteins, fibrinogen was found to be abundant in the silica corona but not albumin, transferrin, or IgG, which could be attributed to its special structure and higher binding affinity to the silica surface. Fibrinogen is a high molecular weight protein (340 kDa) that can bind to a wide range of nanomaterials because it possesses several binding domains that accommodate different nanomaterials.^47^ It has been reported that purified fibrinogen has a higher affinity to the silica surface (plain, amine-, or carboxylic-functionalized NPs) than albumin, transferrin, and IgG.^48^ Soddu et al. has shown that kininogen-1 could have a higher binding affinity to the silica surface than fibrinogen;^30^ however, in this particular low plasma condition, the concentration of kininogen-1 was rather low while the NP surface was more available, which all explain the enrichment of fibrinogen in the studied corona.

Protein precipitation and affinity methods have been used for fibrinogen isolation from human plasma to investigate its N-glycosylation in association with diseases.^49,50^ The former exploits the low solubility of fibrinogen (among the major plasma proteins) and depends on the dehydration of proteins, which likely results in the coprecipitation of fibrinogen with different proteins, particularly albumin.^51^ Both protein precipitation and corona enrichment are not as selective as the affinity method; however, the major advantage of the corona enrichment method for glycan profiling compared to the precipitation method is that it could be utilized in different contexts, for different corona protein enrichment by changing the surface chemistry or plasma protein/NP ratio. The affinity method provided good selectivity, and its application in glycan profiling has been demonstrated.^52^ However, the cost of the antibodies and the optimization steps would need to be considered in a cohort analysis. Furthermore, the corona method could be preferable to the affinity method when the study purpose is to narrow down potential biomarkers by reducing the plasma complexity while providing more details about the glycans of the enriched proteins.

Association between blood clotting disorders and lung cancer has been reported, particularly venous thromboembolism incidents.^53−55^ Fibrinogen is the key component in the coagulation pathway and the degree of sialylation could influence its solubility, hence playing a crucial role in blood clotting processes.^56^ Increased sialylation in fibrinogen has been reported to decrease rates of fibrin polymerization and thinner fibers.^57^ In our lung cancer cohort study, we observed several full plasma N-glycan changes related to the disease, which have been reported elsewhere, including increased sialylation of tri- and tetra-antennary glycans and fucosylation. These changes, however, have also been associated with various disorders, for instance, rheumatoid arthritis, inflammatory bowel disease, and other types of cancer.^14,29^ Interpreting N-glycan profile of full plasma associated with diseases could be challenging as the changes in glycosylation do not always occur in the same direction for all proteins due to differences in the protein biosynthesis routes and plasma concentrations. For example, the concentrations of acute phase proteins, many of them carrying N-glycans, could go up or down depending on specific diseases. As a result, there is a need of targeting specific plasma proteins for glycan-based biomarker discovery. Until now, while IgG glycosylation has been studied extensively for biomarker discovery, the potential application of other plasma proteins remains rather unexplored, partly due to a lack of a simple and cost-efficient enrichment tool for human plasma samples. By exploiting the corona formation and analyzing the glycan profile of a subset of plasma proteins, subtle changes in glycans related to diseases could be observed and attributed to specific enriched proteins. The decrease in 2 glycan peaks containing FA2G2S2 and A3G3S3 observed in the fibrinogen-enriched corona indicates that fibrinogen could actually undergo desialylation, which explains higher blood clotting incidents in the lung cancer patients, while an increase in the global sialylation was observed in the full plasma analysis.

The “gold standard” for glycan profiling, HILIC-HPLC, separates glycans mainly based on their hydrophilicity and degree of branching. It has the capability to separate isomers but not in a complete manner, especially with complex samples.^58^ Reducing the plasma complexity by exploiting biomolecular corona is a feasible option to obtain a higher resolution separation. In addition, changing NP types and exposing conditions would lead to the prefractionation of other glycoproteins. The fibrinogen-enriched corona could also be applied to other diseases, for example, cardiovascular diseases, diabetes, metabolic syndromes, or congenital disorders of glycosylation.

## Conclusions

Biomolecular corona composition is known to vary depending on the types of NPs and biological fluids. In this study, we exploited a specific plasma protein/silica NP concentration ratio to obtain a corona enriched particularly with fibrinogen that was further characterized with physicochemical methods along with glycan profile and proteomics. Overall, the developed method provides a robust and fresh perspective on the application of biomolecular corona to the field of biomarker discovery and a way to evaluate changes in fibrinogen that are associated with chronic diseases. The platform we developed is inexpensive, can be applied in various research laboratories that are not specialized in nanotechnology, and offers an antibody-free approach to prefractionate selective glycoproteins from complex biological matrixes. In addition, different plasma proteins could be enriched by either changing the experimental conditions or the physicochemical properties of the NP of choice. The enrichment method was applied to a small cohort of lung cancer plasma as a proof of concept, and we have successfully identified some glycoproteins and N-glycan peaks, which differed significantly between the lung cancer and non-lung cancer groups.

## Materials and Methods

### Materials

Silica NPs (100 nm, stock concentration of 50 mg/mL) were purchased from Kisker Biotech GmbH (Germany). Phosphate buffer saline (PBS) tablets, Eppendorf LoBind microcentrifuge tubes, trizma base, glycine, acrylamide/bis-acrylamide 40% solution, sodium dodecyl sulfate (SDS), ammonium persulfate (APS), *N*,*N*,*N*′,*N*′-tetramethylethylenediamine (TEMED), ammonium bicarbonate, iodoacetamide, and fibrinogen were purchased from Sigma-Aldrich (Ireland). One PBS tablet was dissolved in 200 mL of ultrapure water to obtain 10 mM PBS (pH 7.4 at 25 °C). Trypsin Gold was purchased from Promega (U.K.). The blue loading buffer pack was purchased from Cell Signaling Technology (Ireland). The BCA kit, C18 pipette tips, and the Imperial protein staining solution were purchased from Thermo Fisher Scientific (TFS, Ireland). The 2D-Silver Stain Reagent II kit was purchased from Cosmo Bio (USA). The Prime-Step prestained protein ladder was purchased from BioLegend (Ireland). The LudgerZyme PNGaseF Kit, LudgerTag Procainamide Glycan Labeling Kit, and Ludger-Clean Procainamide Clean-up Plate were purchased from Ludger Ltd. (U.K.). Human plasma from eight healthy donors provided by the Irish Blood Transfusion Service (IBTS) was mixed in equal proportions to obtain an average pooled plasma. Twenty-five lung cancer plasma [lung adenocarcinoma (15), squamous cell carcinoma (7), small cell lung cancer (1), larger cell lung cancer (1), and lung mesothelioma (1)] and 8 noncancer plasma samples were collected from St. Vincent’s University Hospital Dublin. Eighteen plasma samples of healthy donors were purchased from BioIVT to form the non-lung cancer group with these above 8 noncancer samples. All the plasma sources were prepared from whole blood using coagulant EDTA. Both the pooled plasma and cohort plasma’s total protein concentrations were measured with BCA, following the manufacturer’s instructions. Access and use of plasma samples were covered by the RCSI Ethics number 001246b.

### Silica Corona Sample Preparation

Biomolecular corona samples were prepared by incubating silica NPs with specific plasma concentrations in LoBind tubes. Plasma aliquots were fully defrosted at room temperature and then centrifuged at 16 000 RCF for 3 min to remove any protein aggregations. Plasma solutions were diluted with PBS keeping the final total plasma protein/NP concentration constant and equal to 1.98. The final total volume was 2.0 mL, and the NPs’ concentration was 1.0 mg/mL. NPs were allowed to incubate with the plasma solutions at 37 °C for 1 h with continuous agitation. After the incubation in plasma, the samples were centrifuged for 10 min at 18 000 RCF, room temperature, to pellet the particle–protein complexes and separated from the supernatant plasma. The pellet was then resuspended in 500 μL of PBS and centrifuged again to pellet the biomolecular corona (1 wash). The procedure was repeated 1 and 2 times more to obtain 2 and 3-washed biomolecular coronas, respectively.

### Characterization of Silica Coronas

DLS measurements at θ = 173° were performed using a Zetasizer Nano ZS (Malvern). The sample cuvettes were equilibrated at 25 °C for 90 s. For each measurement, the number of run and duration were automatically determined and repeated three times. Data analysis has been performed according to standard procedures and interpreted through a cumulant expansion of the field autocorrelation function to the second order.

NTA measurements were performed in static mode using a Nanosight NS300 (Malvern) equipped with 488 nm laser. Samples were diluted in PBS to a final volume of 1 mL, so that there were between 30 and 60 nanoparticles/frame. The camera (sCMOS) level was adjusted to have all particles distinctively visible while not saturating the detector. Each sample was recorded 3 times of 60 s each at 25 °C. The sample was manually advanced between the recordings. The videos were analyzed by the built-in NanoSight Software NTA 3.2 using default settings.

Differential centrifugal sedimentation experiments were performed with a CPS Disc Centrifuge DC24000, using the standard sucrose gradient 8–24% (Analytik Ltd.). A PVC calibration standard was used for each sample measurement. The time taken for spherical particles with homogeneous density to travel from the center of the disk to the detector can be directly related to the particle size. Meanwhile, if objects are inhomogeneous, or irregular in shape, the different arrival times still allow one to distinguish between the populations, although their sizes should only be considered as an “apparent” size.^59^ The shell thickness was calculated using the core–shell model.^34^

SDS-PAGE was performed as follows: immediately after the last centrifugation step, the corona pellet was resuspended in protein loading buffer following the manufacturer’s instructions. The washing supernatants were dried down completely in a vacuum concentrator before redispersed in the loading buffer. The samples were boiled for 10 min at 100 °C, and an equal protein amount was loaded into 5–10% fixed polyacrylamide gels. Gel electrophoresis was performed with a Tris-glycine buffer on a Mini-PROTEAN electrophoresis system (Bio-Rad) at a constant voltage of 120 V, for about 60 min, until the proteins neared the end of the gel. The gels were stained with Coomassie blue staining or silver staining, following the manufacturer’s guide. Gels were scanned using an Amersham Imager 600 (GE Healthcare Life Sciences).

### Proteomic LC-MS/MS Sample Preparation and Analysis

Eight MS samples were prepared as previously described.^30^ Non-lung cancer group included plasma samples from 3 healthy donors and 1 individual with negative lung cancer diagnosis. Cancer group consisted of 2 lung adenocarcinoma and 2 squamous cell cancer samples. Samples for each group were randomly picked up while still representing the numbers of sample in the subgroups. Briefly, protein corona samples were run on SDS-PAGE until the blue front line reached the mark at 0.5 cm below the line between the separating and stacking gels. All the area between that line and the blue front containing the sample proteins and its proximity was excised from the gels. The proteins were then fixed, in-gel reduced, and alkylated before digestion with trypsin overnight at 37 °C (14–16 h). The peptide digestion products were then extracted from the gel pieces and cleaned up with the C18 tips, following the manufacturer’s instructions. The peptide amount in each sample was measured with Nanodrop ND-2000 (TFS) before the MS analysis. LC-MS/MS was performed on a Dionex UltiMate3000 nanoRSLC coupled in-line with an Orbitrap Fusion Tribrid mass spectrometer (TFS). Briefly, the peptide samples were loaded onto the trapping column (PepMap100, C18, 300 μm × 5 mm, 5 μm particle size, 100 Å pore size; TFS) for 3 min at a flow rate of 25 μL/min with 2% (v/v) acetonitrile, 0.1% (v/v) trifluoroacetic. Peptides were resolved on an analytical column (Acclaim PepMap 100, 75 μm × 50 cm, 3 μm bead diameter column; TFS) using the following binary gradient; solvent A (0.1% (v/v) formic acid in LC-MS grade water) and solvent B (80% (v/v) acetonitrile, 0.08% (v/v) formic acid in LC-MS grade water) using 3–50% B for 45 min, 50–90% B for 5 min, and holding at 90% B for 5 min at a flow rate of 300 nL/min before returning to 3% B. MS1 spectra were acquired over *m*/*z* 380–1500 in the Orbitrap (120 K resolution at 200 *m*/*z*), and automatic gain control (AGC) was set to accumulate 4 × 10^5^ ions with a maximum injection time of 50 ms. Data-dependent tandem MS analysis was performed using a top-speed approach (cycle time of 3 s), with precursor ions selected in the quadrupole with an isolation width of 1.6 Da. The intensity threshold for fragmentation was set to 5000 and included charge states 2^+^ to 7^+^. Precursor ions were fragmented in the Orbitrap (30 K resolution at 200 *m*/*z*) using higher energy collision dissociation (HCD) with a normalized collision energy of 28%, and the MS2 spectra were acquired with a fixed first *m*/*z* of 110 in the ion trap. A dynamic exclusion of 50 s was applied with a mass tolerance of 10 ppm. AGC was set to 5 × 10^4^ with a maximum injection time set at 300 ms.

Protein identification and quantification were performed with Maxquant, version 1.6.17.0.^60^ Using the Andromeda search engine, the MS/MS spectra were searched against the forward and reverse human Uniprot sequence database, accessed on June 16, 2021 (https://www.uniprot.org). Cysteine carbamidomethylation was set as fixed modification while variable modifications included N-terminal acetylation and methionine oxidation. For both protein and peptide levels, the FDR thresholds were set to 0.01 and only peptides with an amino-acid length of seven or more were considered. The search filtrations were done using a standard target-decoy database approach. Other important search parameters included a value of 0.02 Da for MS/MS mass tolerance, a value of 10 ppm for peptide mass tolerance, and tolerance for the occurrence of up to two missed cleavages. The LFQ was restricted to proteins identified with at least two unique peptides. Additionally, for a protein to be considered valid, two peptide ratios were needed.

Bioinformatic analysis was performed with Perseus software, version 1.6.5.0.^61^ For the pooled silica corona data set, log_2_ summed ion intensities were used to rank proteins, while log_2_ LFQ intensities were used for the cohort protein corona comparison. Imputation of missing values was done by random selection using a normal distribution with a negative shift of 1.8 standard deviations from the mean and with a width of 0.3 standard deviations. These log_2_ LFQ intensities values for all proteins were then used for heatmap presentations (after *z*-scoring) and statistical analysis. Proteome comparisons of the cohort coronas were done with the *t* test, and FDR-corrected *p*-values were used for filtering significant abundance differences. The volcano plot was generated using the default settings (FDR = 0.05, S0 = 0.1). The list of proteins identified in the silica corona was exported to ClueGO/Cytoscape for gene ontology enrichment against *Homo sapiens* organism database.^62^ The ontology Biological Process was selected for the enrichment analysis, and the corrected *p*-values were set to maximal 10^–6^ for the terms to be shown in the DAG.

### Sample Glycan Profiling

In glycan release, the N-glycans were released from the biomolecular corona using a LudgerZyme PNGaseF kit. Briefly, the corona was resuspended in 15 μL of ultrapure water. A volume of 10 μL of 10× denaturation solution was added to each sample and mixed. The samples were incubated for 10 min at 100 °C. The sample tube was briefly vortexed and centrifuged at 18 000 RCF for 10 min to remove NPs. A volume of 20 μL of 10× reaction buffer, 20 μL of 10% NP-40 solution, 135 μL of pure water, and 1 μL of PNGaseF were added to each supernatant containing glycoproteins. Samples were vortexed and incubated overnight at 37 °C (14–16 h).

For fluorescent labeling, 200 μL of each sample was transferred to a nonskirted 96 well PCR plate (300 μL, 4titude Ltd.) and the samples dried down over 9 h. The released N-glycans were converted to aldoses with 40 μL of 0.1% formic acid over 45 min, filtered through a 96-well protein binding plate and dried down completely over 9 h. Released N-glycans were fluorescently labeled by reductive amination with procainamide using a LudgerTag Procainamide Glycan Labeling Kit. Briefly, samples were incubated for 60 min at 65 °C with 20 μL of procainamide labeling solution. The procainamide labeled N glycans were cleaned up using a HILIC-type purification Ludger-Clean Procainamide Clean-up Plate on a vacuum manifold. The purified procainamide labeled N-glycans were eluted with pure water (300 μL).

For LC-ESI-MS and MS/MS analysis, procainamide labeled samples and system suitability standards were analyzed by HILIC-(U)HPLC-ESI-MS with fluorescence detection. To 25 μL of each sample was added 75 μL of acetonitrile. A volume of 25 μL of each sample was injected onto an ACQUITY UPLC BEH-Glycan 1.7 μm, 2.1 mm × 150 mm column (Waters) at 40 °C on an Ultimate 3000 UHPLC instrument with a fluorescence detector (λ_ex_ = 310 nm, λ_em_ = 370 nm), attached to a Bruker Amazon Speed electron-transfer dissociation (ETD) instrument. The chromatography conditions used were solvent A was 50 mM ammonium formate pH 4.4 made from Ludger Stock Buffer, and solvent B was acetonitrile. Gradient conditions were 0 to 53.5 min, 76 to 51% B, 0.4 mL/min; 53.5 to 55.5 min, 51% to 0% B, 0.4 mL/min to 0.2 mL/min; 55.5 to 57.5 min, 0% B at a flow rate of 0.2 mL/min; 57.5 to 59.5 min, 0 to 76% B, 0.2 mL/min; 59.5 to 65.5 min, 76% B, 0.2 mL/min; 65.5 to 66.5 min, 76% B, 0.2 mL/min to 0.4 mL/min; 66.5 to 70.0 min, 76% B, 0.4 mL/min. The Amazon Speed settings were source temperature, 250 °C; gas flow, 10 L/min; capillary voltage, 4500 V; ICC target, 200 000; max accu time, 50.00 ms; rolling average, 2; number of precursor ions selected, 3; release after 0.2 min; positive ion mode; scan mode, enhanced resolution; mass range scanned, 500–1700; target mass, 900.

Glycan structures were assigned with Bruker Compass DataAnalysis and GlycoWorkbench 2 software.^63^ The glycan structure compositions were identified by using the registered parent *m*/*z* values from the full MS scan. Potential glycan structures were then in-silico defragmented to generate their theoretical ion *m*/*z*. The calculated and registered *m*/*z* values from the MS/MS scan were then compared to confirm the presence of the structures. Peak integration was performed with HappyTools that did the peak calibration and integration by examining user-defined calibrant and analyte peak lists, respectively.^46^ For the calibration, we used 4–5 glycan peaks with high signal-to-noise ratios that were spaced out roughly equally in the chromatograms. The analyses were performed in two separate batches, one for all the full plasma chromatograms and the other for all silica corona chromatograms. Relative abundances of the peaks were obtained directly from the software outputs.

### Statistics and Data Plotting

Statistical analysis was performed in R Studio v1.1.463 (the R Foundation for Statistical Computing) running R version 4.0.4. Relative peak areas under the curve were log-transformed [log (1/peak – 1)] and the normality of distribution was determined using the Shapiro–Wilk test. Normally and non-normally distributed data were compared using two-tailed Student’s *t* test and Mann–Whitney’s U-tests, respectively. Associations between sex and each log-transformed peak area were compared in univariate pairwise analyses. Associations between age and individual log-transformed peak areas were examined visually by a scatter plot in the first instance and then with generalized linear models incorporating sex and age as covariates. To correct for multiple testing, *p*-values in the pairwise analyses were corrected using the Bonferroni method and were considered significant if <0.05.

Other data were analyzed and plotted with ImageJ version 1.53c (Fiji package version 2.1.0), GraphPad Prism (version 9), and Excel (Office 2016). The abstract figure was made with ChemDraw (version 16.0).

## Supporting Information Available

Additional data on the biomolecular corona protocol optimization and characterization, the full plasma protein and protein corona variability across eight different donors, the protein corona grouping obtained from the mass spectrometry analysis, along with the glycan analysis of the full plasma and corona samples (PDF)
Glycan assignment of the full plasma glycan structures, assignment of glycan structures of silica corona, the glycan composition changes of peaks with the same retention times after plasma protein enrichment, the statistical test of full plasma glycan peaks, and the statistical test of silica corona glycan peaks (XLSX)
