# Supplementary material for: Nanoparticle Biomolecular Corona-Based Enrichment of Plasma Glycoproteins for N-Glycan Profiling and Application in Biomarker Discovery
Source: ACS Nano. 2022 Mar 28;16(4):5463–75. doi: 10.1021/acsnano.1c09564 (PMC9047655; doi:10.1021/acsnano.1c09564)
Supplement: Supplementary file 1 — nn1c09564_si_001.pdf [file nn1c09564_si_001.pdf]

Supporting Information

Nanoparticle biomolecular corona-based enrichment of plasma glycoproteins for N-glycan profiling and application in biomarker discovery

Duong N. Trinh [1], Richard A. Gardner [2], Alessandro N. Franciosi [3], Cormac McCarthy [4,5], Michael P. Keane [4,5], Mahmoud G. Soliman [1,6], James S. O'Donnell [7], Paula Meleady [8], Daniel I. R. Spencer [2], Marco P. Monopoli [1,*]

1 Department of Chemistry, Royal College of Surgeons in Ireland, University of Medicine and Health Sciences, Dublin 2, Ireland

2 Ludger Ltd, Culham Science Centre, Abingdon, Oxfordshire, United Kingdom, OX14 3EB

3 UBC Faculty of Medicine, Department of Respiratory Medicine, University of British Columbia, Vancouver, British Columbia, Canada, V6Z 1Y6

4 Department of Respiratory Medicine, St. Vincent's University Hospital, Dublin 4, Ireland.

5 School of Medicine, University College Dublin, Dublin 4, Ireland

6 Physics Department, Faculty of Science, Al-Azhar University, Nasr City 11884, Cairo, Egypt

7 Irish Centre for Vascular Biology, School of Pharmacy and Biomolecular Sciences, Royal College of Surgeons in Ireland, University of Medicine and Health Sciences, Dublin 2, Ireland

8 School of Biotechnology, Dublin City University, Dublin 9, Ireland

*** Corresponding authors**

E-mail: marcomonopoli@rcsi.ie

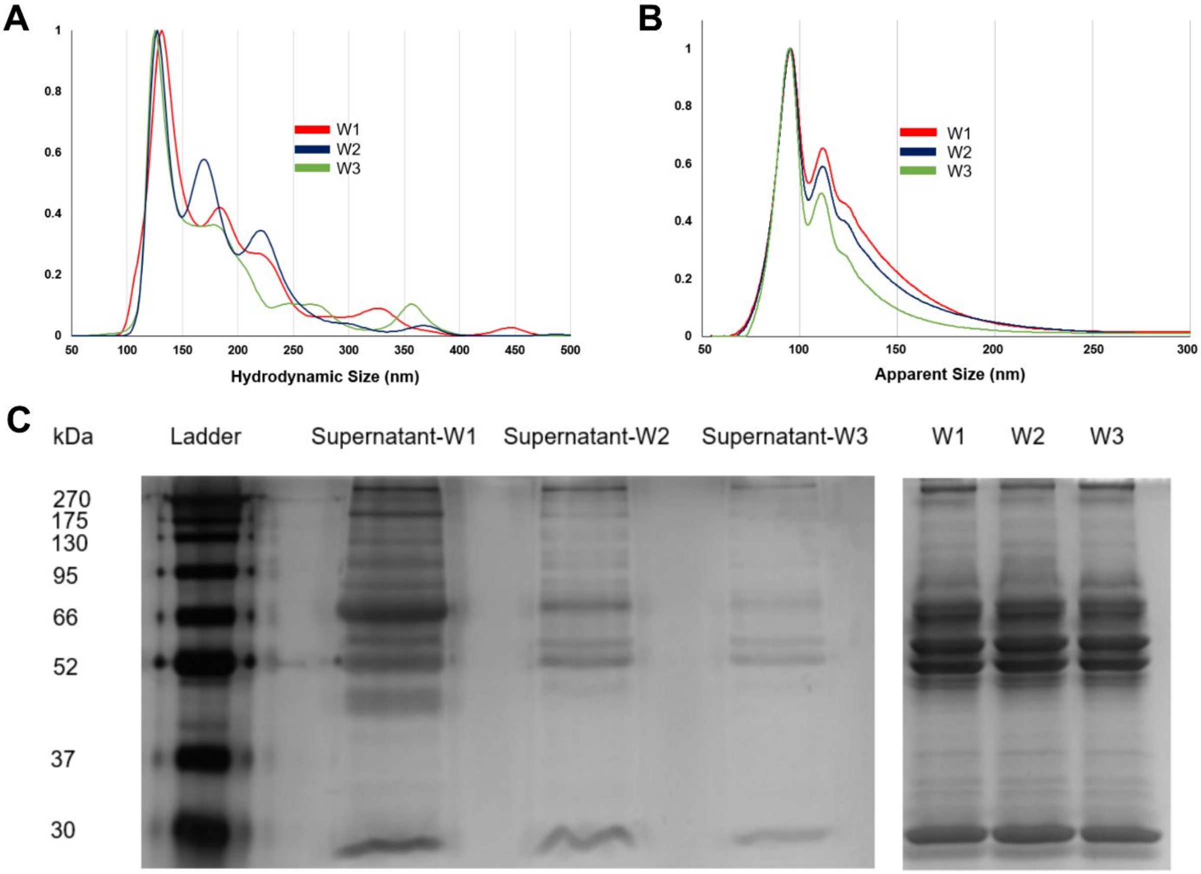

Figure S1: Size distribution and protein composition of the silica coronas after different numbers of washes. A) NTA hydrodynamic size distributions of silica corona, 1, 2 and 3 washes (W1, W2 and W3). The data are shown as the average of 3 measurements. The peaks were normalised against the tallest peaks (highest particle concentration) that had a value of 1. B) DCS size distributions of silica corona, 1, 2 and 3 washes (W1, W2 and W3). The data were shown as relative weight particle size distribution. The tallest peak (highest weight value) had a value of 1 and all other particle size peaks were then normalized against this base peak to give a relative weight distribution. C) Protein loss from the silica corona after 1, 2 and 3 washes (supernatant-W1, supernatant-W2 and supernatant-W3) and the corresponding coronas after 1, 2 and 3 washes (W1, W2 and W3).

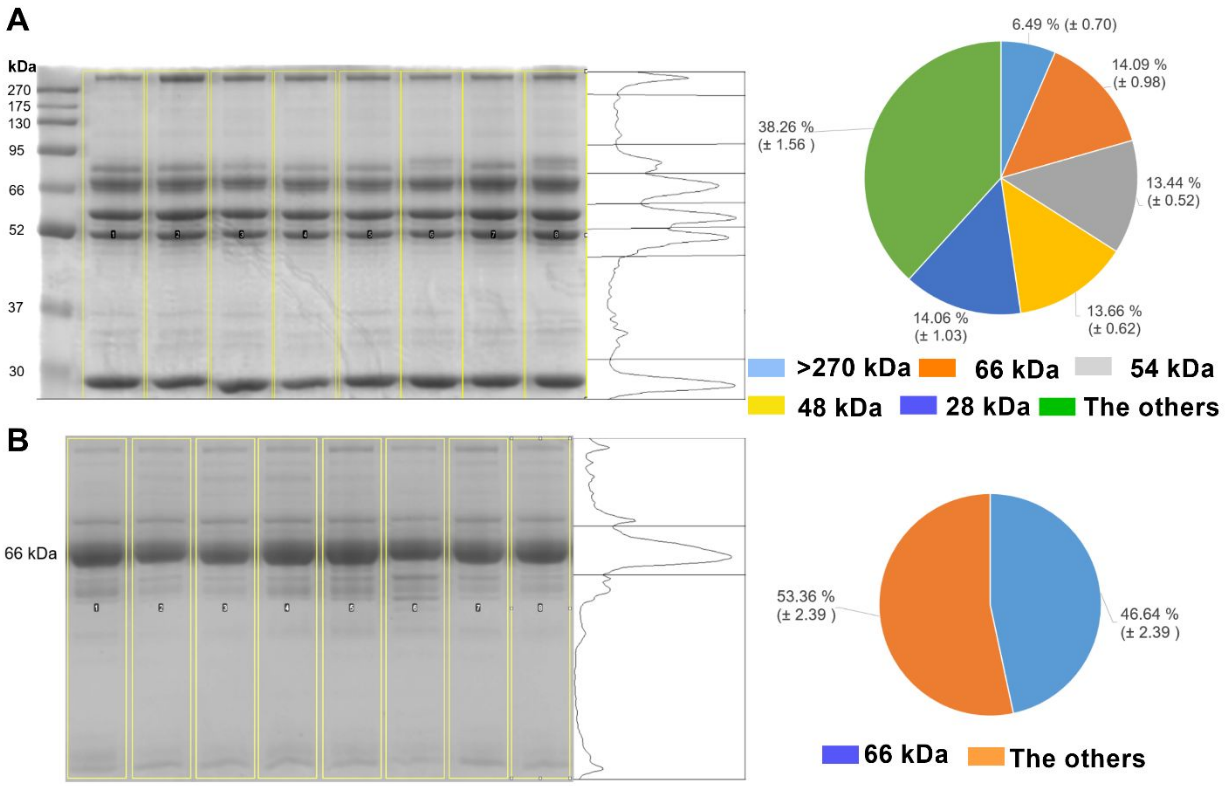

Figure S2: Gel densitometry and the intensity percentages of proteins bands in the silica corona (B) and full plasma obtained from 8 healthy donors (C). The band intensities normalised by total were shown in the corresponding pie charts with their standard deviation (n = 8).

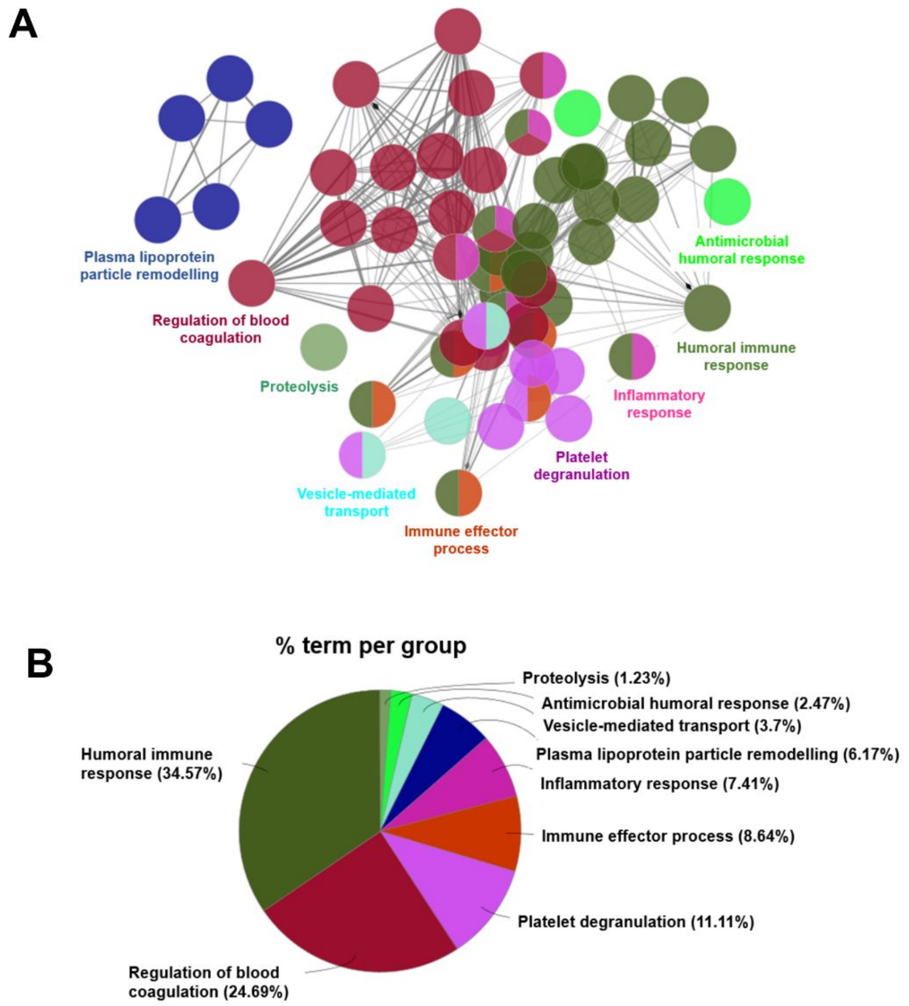

Figure S3: Enrichment analysis of silica corona proteins. A) Directed acyclic graph (DAG) shows the GO enrichment based on the Biological Process term. B) The term percentages of nine enriched protein biological process groups.

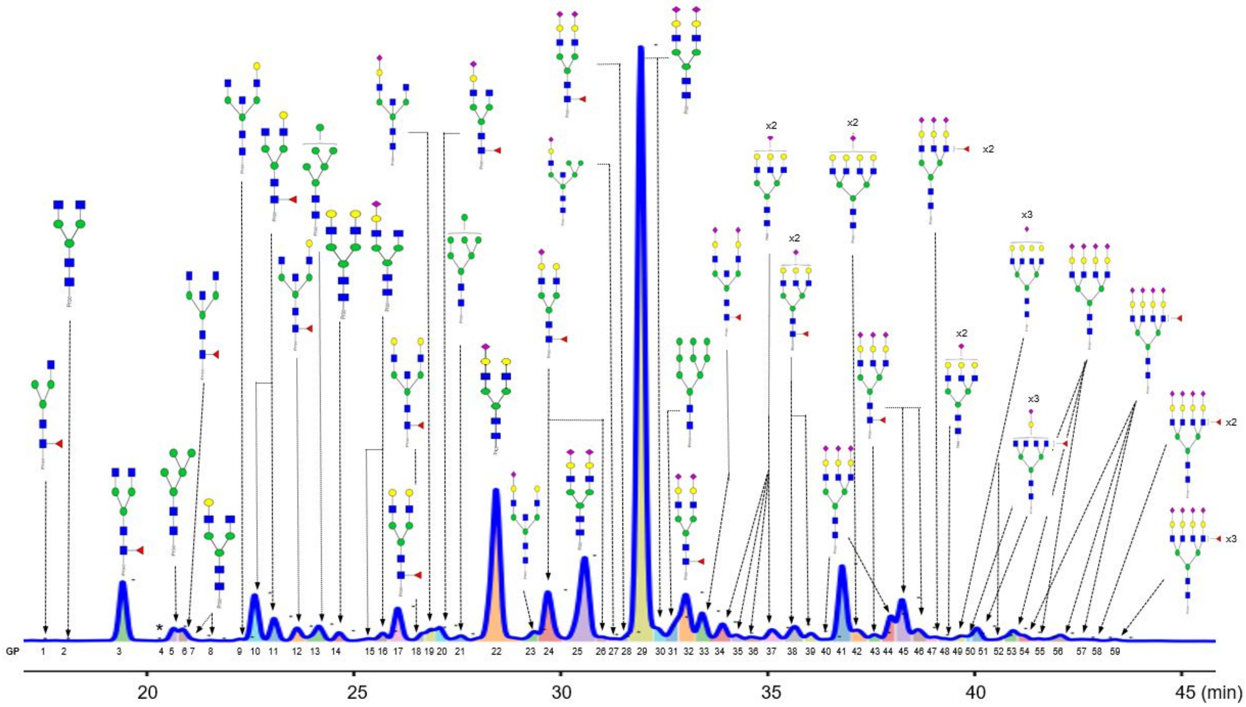

Figure S4: N-glycan profile of the full plasma. The glycan linkages of galactose, fucose and sialic acid are not specified. For a peak with multiple glycan structures detected, only the major structure is shown. *N*-acetylglucosamine (blue square), fucose (red triangle), mannose (green circle), galactose (yellow circle) and *N*-acetylneuraminic acid (purple diamond). *: GP4 contains a contaminant structure, whose structure is not shown in the figure.

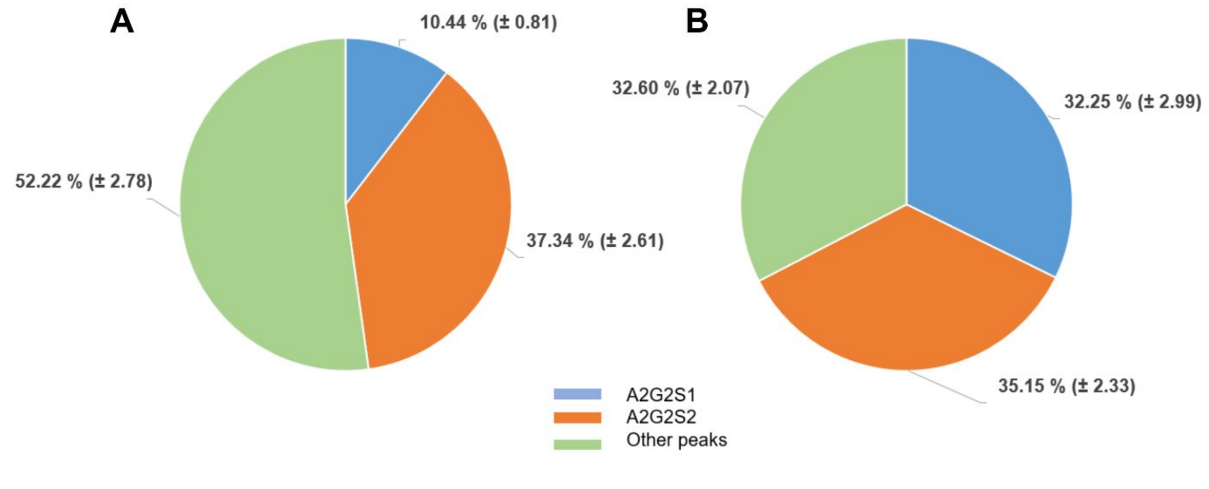

Figure S5: Normalised areas under the curve (AUCs) of glycan peaks A2G2S1 and A2G2S2 in comparison with all other peaks. The peak intensity percentages in the full plasma (A) and silica corona (B) obtained from healthy donors are shown with their standard deviation (n = 8).

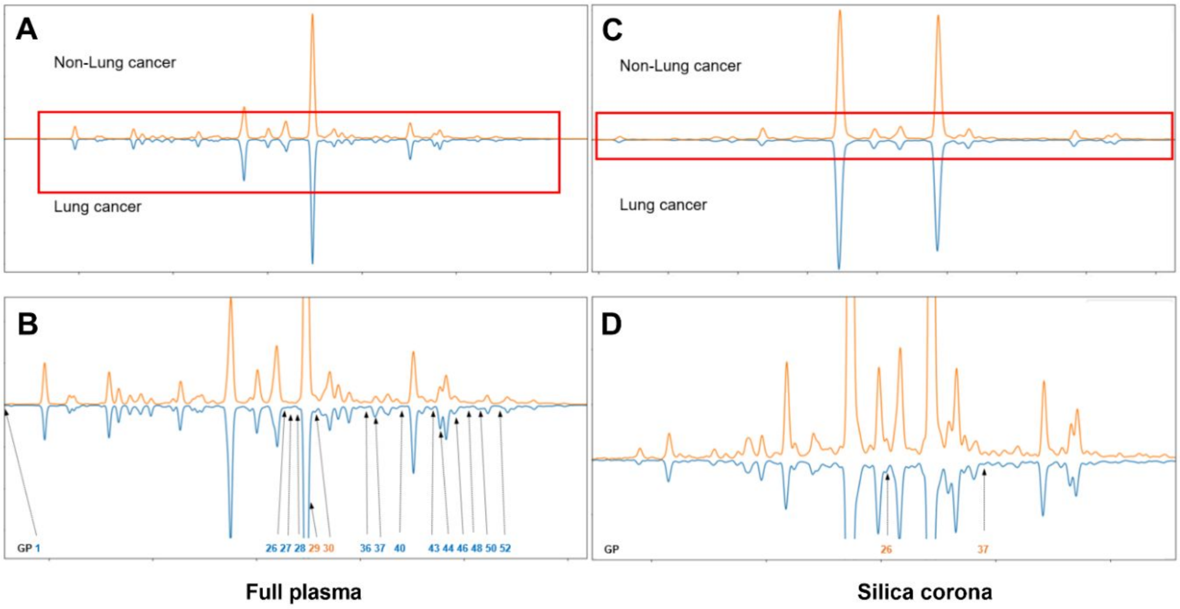

Figure S6: Glycan profiles of full plasma (A-B) and silica corona (C-D), comparing the lung cancer and non-lung cancer groups. A) The chromatograms of released glycans from full plasma of a non-lung cancer sample (up, orange) and lung cancer sample (down, blue) were normalised to the highest peak intensity ($GP_{corona}31$). B) The zoomed-in section of the box highlighted in Figure A. Only peaks with significant differences are shown. The filled colour of the numbers match that of the chromatogram, in which they were more abundant. C) The chromatograms of released glycans from silica corona of a non-lung cancer sample (up, orange) and lung cancer sample (down, blue) were normalised to the highest peak intensity ($GP_{corona}22$). D) The zoomed-in section of the box highlighted in Figure C showing significant peaks.
